# An efficient synthesis of 1,6-anhydro-*N*-acetylmuramic acid from *N*-acetylglucosamine

**DOI:** 10.3762/bjoc.13.261

**Published:** 2017-12-11

**Authors:** Matthew B Calvert, Christoph Mayer, Alexander Titz

**Affiliations:** 1 Chemical Biology of Carbohydrates, Helmholtz Institute for Pharmaceutical Research Saarland (HIPS), D-66123 Saarbrücken, Germany; 2 Deutsches Zentrum für Infektionsforschung (DZIF), Standort Hannover-Braunschweig, Germany; 3 Interfaculty Institute of Microbiology and Infection Medicine Tübingen (IMIT), Department of Microbiology and Biotechnology, University of Tübingen, Germany; 4 Department of Pharmacy, Saarland University, Saarbrücken, Germany

**Keywords:** *N*-acetylmuramic acid, anhydrosugars, antibiotic resistance, bacterial cell wall recycling, carbohydrate synthesis

## Abstract

A novel synthesis of 1,6-anhydro-*N*-acetylmuramic acid is described, which proceeds in only five steps from the cheap starting material *N*-acetylglucosamine. This efficient synthesis should enable future studies into the importance of 1,6-anhydromuramic acid in bacterial cell wall recycling processes.

## Introduction

**Figure 1:**
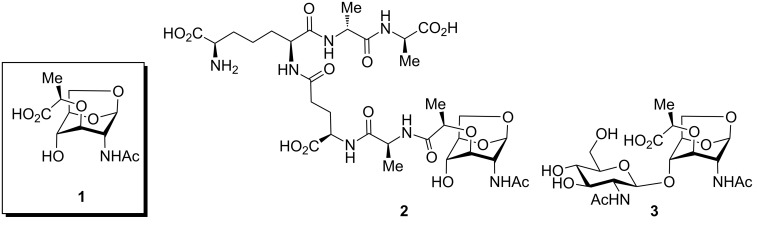
AnhydroMurNAc and derivatives.

1,6-Anhydro-*N*-acetylmuramic acid (AnhydroMurNAc, **1**, Figure 1) and its derivatives (e.g., **2** and **3**) are of great interest due to their integral role in bacterial cell wall recycling [1–2] and the induction of antibacterial resistance [3]. Numerous syntheses of **1** have been carried out, including early synthetic work by Sinaÿ [4–5] and Paulsen [6–7], and more recent syntheses by Mobashery [8], Fukase [9], and Mark [10], which have enabled further studies in the field of antibacterial research.

**Scheme 1:**
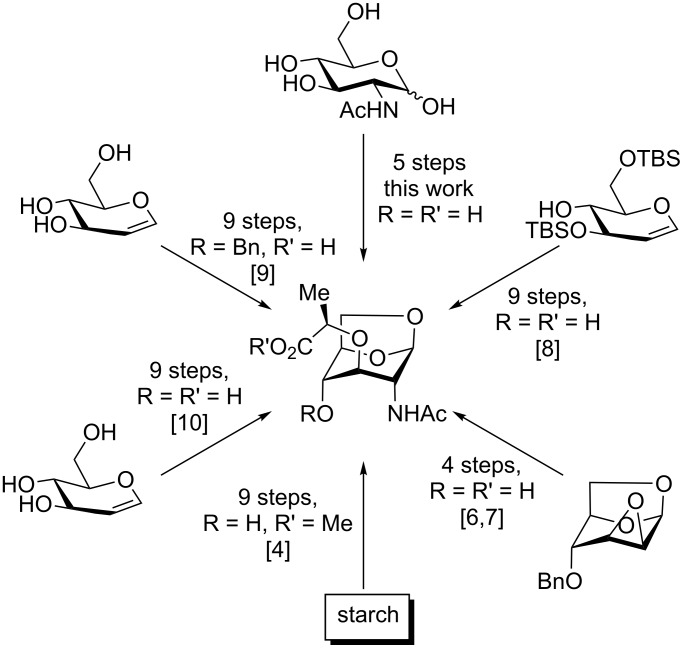
Our approach to AnhydroMurNAc compared with literature preparations.

Looking to access significant quantities of **1**, we were dissatisfied with the existing routes to this relatively simple molecule, which require many steps and/or expensive starting materials (Scheme 1). As such, we investigated a shorter synthesis of the target molecule starting from cheap *N*-acetylglucosamine (≈0.1 €/g).

## Results and Discussion

**Scheme 2:**
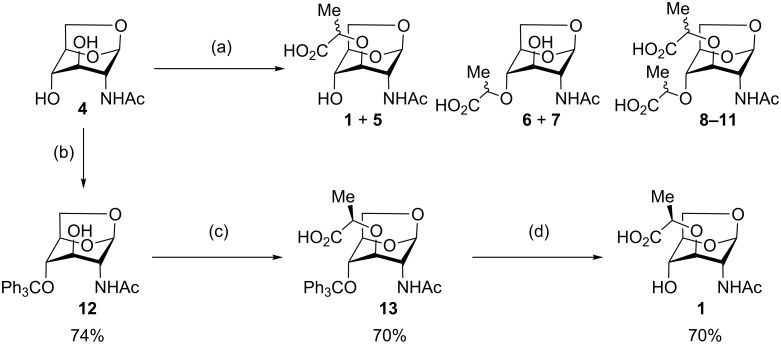
Synthesis of AnhydroMurNAc. Conditions: (a) (±)-2-chloropropionic acid, NaH, dioxane, 45 °C to 90 °C, 2 h; (b) TrOTf, collidine, CH_2_Cl_2_, rt, 2 h; (c) (*S*)-2-chloropropionic acid, NaH, dioxane, 45 °C to 90 °C, 30 min; (d) trifluoroacetic acid, CHCl_3_, 0 °C to rt, 3 h.

1,6-AnhydroGlcNAc (2-acetamido-1,6-anhydro-2-deoxy-α-D-glucopyranoside, **4**, Scheme 2) can be prepared in two steps from GlcNAc [11], and this molecule was thus used as the starting point for our synthetic studies. Initial attempts to alkylate anhydroGlcNAc **4** directly gave a complex mixture of stereo- and regioisomers, out of which the desired anhydroMurNAc derivative **1** could only be isolated in trace quantities from the mixture with isomers **5**–**11** (Scheme 2). A selective protection of the 4-OH position of **4** has been reported, and as such this tritylation was carried out, delivering **12** in 74% yield [12]. The desired alkylation step provides **13** in good yield and excellent diastereoselectivity using commercially available (*S*)-2-chloropropionic acid as the alkylating agent. Trityl deprotection could be readily accomplished without the need to protect the carboxylic acid, delivering the target compound **1** in good yield and high purity after work-up without the need for chromatographic purification.

**Figure 2:**
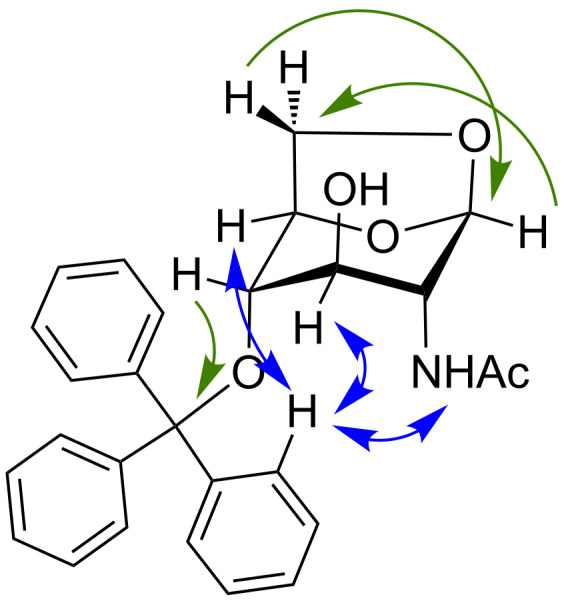
2D NMR correlations confirming the structure of **12**.

It should be noted that there were some surprising differences between the NMR data reported for compound **12** in CDCl_3_ and that which we obtained, with dissimilar proton shifts observed for the acetyl group (2.198 reported vs 2.00 observed) and one of the H6 protons (4.350 reported vs 4.03 observed) [12]. However, when 2D NMR studies were carried out in acetone-*d*_6_, in which **12** is significantly more stable, we were able to unequivocally prove the reported structure by identifying key HMBC correlations between H-1 and C-6, and between H-4 and the quarternary trityl carbon (Figure 2, green single-headed arrows). NOESY correlations between the trityl group and the GlcNAc protons on the lower face of the ring further confirmed the expected structure (Figure 2, blue double-headed arrows; see Supporting Information File 1 for further details).

**Scheme 3:**
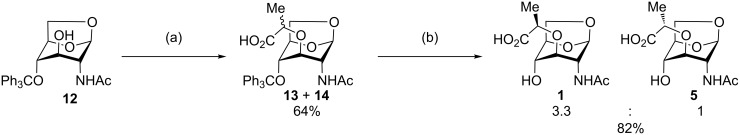
Synthesis of AnhydroMurNAc and diastereomer **5**. Conditions: (a) (±)-2-chloropropionic acid, NaH, dioxane, 45 °C to 90 °C, 3 h; (b) trifluoroacetic acid, CHCl_3_, 0 °C to rt, 3 h.

Interestingly, when the alkylation of **12** was carried out using racemic 2-chloropropionic acid, a 3.3:1 mixture of diastereomers **13** and **14** was obtained, favouring the desired diastereomer **13** (Scheme 3). The diastereomers were separable by HPLC following trityl deprotection, enabling access to the unnatural isomuramic acid derivative **5**.

## Conclusion

In conclusion, a novel approach to anhydroMurNAc has been devised. The main advantage of this approach is the short synthetic sequence, which provides access to the target compound in only five steps from the cheap starting material *N*-acetylglucosamine.

## Experimental

### General details

Silica gel 60-coated aluminum sheets containing fluorescence indicator (Macherey-Nagel, Düren, Germany) were used for thin-layer chromatography (TLC). UV light (254 nm) and vanillin solution (0.4 M solution of vanillin in 1% ethanolic H_2_SO_4_) or molybdate solution (0.02 M solution of ammonium cerium sulfate dihydrate and ammonium molybdate tetrahydrate in 10% aqueous H_2_SO_4_) were used for development. Preparative medium pressure liquid chromatography (MPLC) was performed on a Teledyne Isco Combiflash Rf200 system using pre-packed silica gel 60. Optical rotation was measured using a P-2000 polarimeter (Jasco, Gross-Umstadt, Germany) at 589 nm. Nuclear magnetic resonance (NMR) spectroscopy was performed on a Bruker Avance III 500 UltraShield spectrometer at 500 MHz (^1^H) or 126 MHz (^13^C) or a Bruker Fourier 300 spectrometer at 300 MHz (^1^H) or 75 MHz (^13^C). Chemical shifts are given in ppm and were calibrated on residual solvent peaks as internal standard [13]. Multiplicities were specified as s (singlet), d (doublet), t (triplet) or m (multiplet). The signals were assigned with the help of ^1^H,^1^H-COSY, DEPT-135-edited ^1^H,^13^C-HSQC, ^1^H,^13^C-HMBC, and NOESY experiments. Mass spectra were obtained on a Bruker amaZon SL for low resolution or on a Bruker maXis 4G HR-QTOF spectrometer for high resolution, and the data were analyzed using DataAnalysis (Bruker Daltonics, Bremen, Germany). High-pressure liquid chromatography (HPLC) was carried out on a Macherey-Nagel Nucleodur^®^ 250/21 C18 column. Commercial chemicals and solvents were used without further purification. Deuterated solvents were purchased from Eurisotop (Saarbrücken, Germany) or Deutero (Kastellaun, Germany).

### 2-Acetamido-1,6-anhydro-2-deoxy-α-D-glucopyranose (**4**)


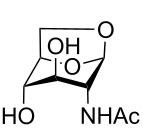


The synthesis of 1,6-anhydroGlcNAc was carried out according to Lafont et al. [11]. ^1^H NMR (300 MHz, methanol-*d*_4_) δ 5.26 (s, 1H, H-1), 4.49 (d, *J* = 5.5 Hz, 1H, H-5), 4.18 (dd, *J* = 7.2, 1.0 Hz, 1H, H-6a), 3.83 (s, 1H, H-2), 3.68 (dd, *J* = 7.1, 5.7 Hz, 1H, H-6b), 3.58–3.52 (m, 2H, H-3 + H-4), 1.98 (s, 3H, Me); ^13^C NMR (75 MHz, MeOD) δ 172.61 (C=O), 102.29 (CH, C-1), 77.74 (CH, C-5), 73.36 (CH, C-3), 72.54 (CH, C-4), 66.38 (CH_2_, C-6), 53.61 (CH, C-2), 22.63 (Me). In agreement with literature data [14].

### 4-*O*-Triphenylmethyl-2-acetamido-1,6-anhydro-2-deoxy-α-D-glucopyranose (**12**)


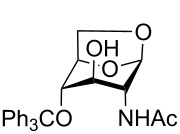


The synthesis of 4-trityl-1,6-anhydroGlcNAc was carried out according to Tyrtysh et al. [12].

A suspension of **4** (203 mg, 1.0 mmol) in dichloromethane (10 mL) was treated with collidine (264 µL, 2.0 mmol) at room temperature. Trityl triflate (0.3 M in dichloromethane, 5 mL, generated freshly by mixing equimolar quantities of TMSOTf and TrOH) was added over five minutes, with further collidine (264 µL) and TrOTf (0.3 M, 5 mL) added portionwise over two hours. The reaction mixture was diluted with pyridine (300 µL) and methanol (500 µL), followed by the addition of chloroform (80 mL). The solution was washed with water, HCl (1 M), water, NaHCO_3_ (saturated aqueous solution) and water (50 mL each). The organic layer was dried (Na_2_SO_4_), filtered, and concentrated in vacuo. Purification by MPLC (0–5% MeOH in 1:9 petroleum ether/chloroform) yielded the title compound (330 mg, 0.74 mmol, 74%) as a colourless solid. ^1^H NMR (500 MHz, acetone-*d*_6_) δ 7.60–7.52 (m, 6H), 7.38–7.33 (m, 6H), 7.31–7.26 (m, 3H), 6.85 (d, *J* = 8.9 Hz, 1H, NH), 5.20 (s, 1H, H-1), 4.43 (d, *J* = 4.2 Hz, 1H, OH), 3.93 (dd, *J* = 7.1, 1.1 Hz, 1H, H-6a), 3.73 (d, *J* = 8.8 Hz, 1H, H-2), 3.71 (d, *J* = 5.9 Hz, 1H, H-5), 3.68–3.63 (m, 1H, H-4), 3.39–3.33 (m, 1H, H-3), 3.29 (dd, *J* = 7.2, 6.1 Hz, 1H, H-6b), 1.97 (s, 3H, Ac); ^13^C NMR (126 MHz, acetone-*d*_6_) δ 169.56 (C=O, Ac), 145.34 (3 × C), 129.67 (6 × CH), 128.81 (6 × CH), 128.18 (3 × CH), 101.63 (CH, C-1), 88.72 (C), 76.00 (CH, C-5), 74.55 (CH, C-4), 71.73 (CH, C-3), 65.85 (CH_2_, C-6), 53.47 (CH, C-2), 23.09 (Me, Ac).

### 4-*O*-Triphenylmethyl-1,6-anhydro-*N*-acetylmuramic acid (**13**)


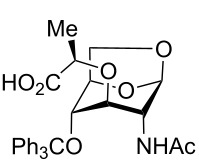


A solution of **12** (910 mg, 2.0 mmol) in dioxane (16 mL) was treated with sodium hydride (60% suspension in mineral oil, 540 mg, 13.5 mmol) at room temperature. The suspension was warmed to 45 °C for 10 minutes, then cooled to rt. (*S*)-2-Chloropropionic acid (540 mg, 450 µL, 5.0 mmol) was added, and the suspension stirred at 90 °C for 30 minutes. The reaction mixture was concentrated in vacuo, treated dropwise with ice-water (20 mL), acidified to pH 3 at 0 °C with HCl (2.5 M) and extracted exhaustively with dichloromethane. The pooled organics were dried (Na_2_SO_4_), filtered, and concentrated in vacuo. Purification by MPLC (5% to 30% (1:9 acetic acid/ethyl acetate) in dichloromethane) yielded the title compound (720 mg, 1.4 mmol, 70%) as a colourless solid, 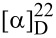 −43.0 (*c* 1.15, CHCl_3_); ^1^H NMR (300 MHz, DMSO-*d*_6_) δ 12.37 (s, 1H, COOH), 7.71 (d, *J* = 7.7 Hz, 1H, NH), 7.48 (dt, *J* = 6.2, 1.5 Hz, 6H, 6 × ArH), 7.40–7.26 (m, 9H, 9 × ArH), 5.23 (d, *J* = 1.7 Hz, 1H, H-1), 3.80–3.64 (m, 3H, H-6a, Lac-α, H-2), 3.55 (d, *J* = 5.8 Hz, 1H, H-5), 3.50 (s, 1H, H-4), 3.31 (s, 1H, H-3), 3.23 (t, *J* = 6.6 Hz, 1H, H-6b), 1.94 (s, 3H, Me, Ac), 1.07 (d, *J* = 6.7 Hz, 3H, Me, Lac-Me); ^13^C NMR (75 MHz, DMSO-*d*_6_) δ 173.54 (C=O, COOH), 168.83 (C=O, Ac), 144.06 (3 × C), 128.53 (6 × CH), 128.04 (6 × CH), 127.38 (3 × CH), 99.87 (CH, C-1), 87.54 (C, CPh3), 77.14 (CH, C-3), 74.04 (CH, C-5), 72.76 (CH, Lac-α), 71.62 (CH, C-4), 64.48 (CH_2_, C-6), 49.87 (CH, C-2), 22.59 (Me, Ac), 18.14 (Me, Lac-Me); HRMS (ESI–TOF) *m/z*: [M + H]^+^ calcd for C_30_H_32_NO_7_, 518.21788; found, 518.21596.

### 1,6-Anhydro-*N*-acetylmuramic acid (**1**)


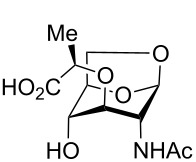


A solution of **13** (720 mg, 1.4 mmol) in chloroform (20 mL) was treated with trifluoroacetic acid (1.0 mL) at 0 °C. The reaction mixture was allowed to warm to room temperature, stirred for 3 hours and then coevaporated with toluene (100 mL). The residue was diluted with diethyl ether and water (25 mL each), the layers separated and the aqueous layer further extracted with diethyl ether (2 × 25 mL). The aqueous layer was lyophilised to provide the title compound (270 mg, 1.0 mmol, 70%) as a colourless solid, 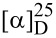 −11.4 (*c* 1.0, water); ^1^H NMR (500 MHz, acetonitrile-*d*_3_) δ 6.60 (d, *J* = 8.4 Hz, 1H, NH), 5.31 (s, 1H, H-1), 4.49 (d, *J* = 5.1 Hz, 1H, H-5), 4.24 (q, *J* = 6.8 Hz, 1H, α-Lac), 4.11 (dd, *J* = 7.5, 1.0 Hz, 1H, H-6a), 3.87 (dd, *J* = 8.7, 1.7 Hz, 1H, H-2), 3.70–3.63 (m, 2H, H-4, H-6b), 3.41–3.34 (m, 1H, H-3), 1.91 (s, 3H, Ac), 1.36 (d, *J* = 6.8 Hz, 3H, Lac-Me); ^13^C NMR (126 MHz, CD_3_CN) δ 174.70 (C=O, COOH), 170.82 (C=O, Ac), 101.69 (CH, C-1), 80.03 (CH, C-3), 77.32 (CH, C-5), 74.94 (CH, α-Lac), 69.98 (CH, C-4), 66.19 (CH_2_, C-6), 50.26 (CH, C-2), 23.05 (Me, Ac), 18.65 (Me, Lac-Me). NMR data in agreement with literature values [8]. HRMS (ESI–TOF) *m/z*: [M + H]^+^ calcd for C_11_H_18_NO_7_, 276.10833; found, 276.10892.

### 4-*O*-Triphenylmethyl-1,6-anhydro-*N*-acetylmuramic acid (**13**) and 4-*O*-triphenylmethyl-1,6-anhydro-*N*-acetylisomuramic acid (**14**)


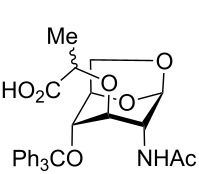


A solution of **12** (224 mg, 0.5 mmol) in dioxane (2.5 mL) was treated with sodium hydride (60% suspension in mineral oil, 130 mg, 3.3 mmol) at room temperature. The suspension was warmed to 45 °C for 10 minutes, then cooled to rt. (±)-2-Chloropropionic acid (130 mg, 110 µL, 1.2 mmol) was added, and the suspension stirred at 90 °C for 3 hours. The reaction mixture was concentrated in vacuo, treated dropwise with ice-water (5 mL), acidified to pH 3 at 0 °C with HCl (2.5 M) and extracted exhaustively with dichloromethane. The pooled organics were dried (Na_2_SO_4_), filtered, and concentrated in vacuo. Purification by MPLC (10% to 30% (1:9 acetic acid/ethyl acetate) in dichloromethane) yielded the title compound (165 mg, 0.32 mmol, 64%) as a mixture of diastereomers which were separated following trityl deprotection.

### 1,6-Anhydro-*N*-acetylmuramic acid (**1**) and 1,6-anhydro-*N*-acetylisomuramic acid (**5**)


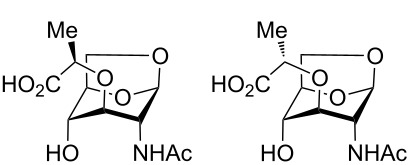


A solution of **13** and **14** (52 mg, 0.1 mmol) in chloroform (10 mL) was treated with trifluoroacetic acid (0.1 mL) at 0 °C. The reaction mixture was allowed to warm to room temperature, stirred for 3 hours and then coevaporated with toluene (2 × 10 mL). The residue was purified by HPLC (5% acetonitrile + 0.1% formic acid, 9.5 mL/min) to provide the title compounds. Muramic acid derivative **1** (17.4 mg, 63 µmol, 63%): Colourless solid. Analytical data as above.

Isomuramic acid derivative **5** (5.2 mg, 19 µmol, 19%): Colourless solid, 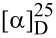 −86.6 (*c* 1.0, water); ^1^H NMR (500 MHz, acetonitrile-*d*_3_) δ 6.62 (d, *J* = 9.0 Hz, 1H, NH), 5.23 (s, 1H, H-1), 4.44 (d, *J* = 5.1 Hz, 1H, H-5), 4.24 (q, *J* = 6.9 Hz, 1H, α-Lac), 4.10 (dd, *J* = 7.2, 1.1 Hz, 1H, H-6a), 3.75 (dd, *J* = 9.0, 1.6 Hz, 3H, H-2), 3.68 (s, 1H, H-4), 3.61 (dd, *J* = 7.2, 5.8 Hz, 1H, H-6b), 3.27 (s, 1H, H-3), 1.90 (s, 3H, Ac), 1.33 (d, *J* = 6.9 Hz, 3H, Lac-Me); ^13^C NMR (126 MHz, acetonitrile-*d*_3_) δ 175.43 (C=O, COOH), 170.91 (C=O, Ac), 101.81 (CH, C-1), 80.20 (CH, C-3), 77.16 (CH, C-5), 74.58 (CH, α-Lac), 70.94 (CH, C-4), 65.97 (CH_2_, C-6), 50.97 (CH, C-2), 23.05 (Me, Ac), 19.23 (Me, Lac-Me); HRMS (ESI–TOF) *m/z*: [M + H]^+^ calcd for C_11_H_18_NO_7_, 276.10833; found, 276.10713.

## Supporting Information

File 1 NMR spectra.
