# Supplementary material for: An efficient synthesis of 1,6-anhydro-N-acetylmuramic acid from N-acetylglucosamine
Source: Beilstein J Org Chem. 2017 Dec 11;13:2631–6. doi: 10.3762/bjoc.13.261 (PMC5753053; doi:10.3762/bjoc.13.261)
Supplement: File 1 — NMR spectra. [file Beilstein_J_Org_Chem-13-2631-s001.pdf]

Supporting Information

for

An efficient synthesis of 1,6-anhydro-*N*-acetylmuramic acid from *N*-acetylglucosamine

Matthew B. Calvert[1,2], Christoph Mayer[3], Alexander Titz[1,2,4*]

Address: [1]Chemical Biology of Carbohydrates, Helmholtz Institute for Pharmaceutical Research Saarland (HIPS), D-66123 Saarbrücken, Germany, [2]Deutsches Zentrum für Infektionsforschung (DZIF), Standort Hannover-Braunschweig, Germany, [3]Interfaculty Institute of Microbiology and Infection Medicine Tübingen (IMIT), Department of Microbiology and Biotechnology, University of Tübingen, Germany and [4]Department of Pharmacy, Saarland University, Saarbrücken, Germany

Email: Alexander Titz - alexander.titz@helmholtz-hzi.de *Corresponding author

Contents

Table of Contents	S1
**Confirming the reported structure of 12**	S2
^{1}H NMR data for **12** in $CDCl_3$, table of observed and reported proton shifts	S2
HMBC data for **12** (acetone-d_6) with key correlations indicated	S3
NOESY data for **12** (acetone-d_6) with key correlations indicated	S4
**^{1}H and ^{13}C NMR data**	S5
^{1}H NMR data for **12**	S5
^{13}C NMR data for **12**	S6
^{1}H NMR data for **13**	S7
^{13}C NMR data for **13**	S8
^{1}H NMR data for **1**	S9
^{13}C NMR data for **1**	S10
^{1}H NMR data for **5**	S11
^{13}C NMR data for **5**	S12

Confirming the reported structure of **12**

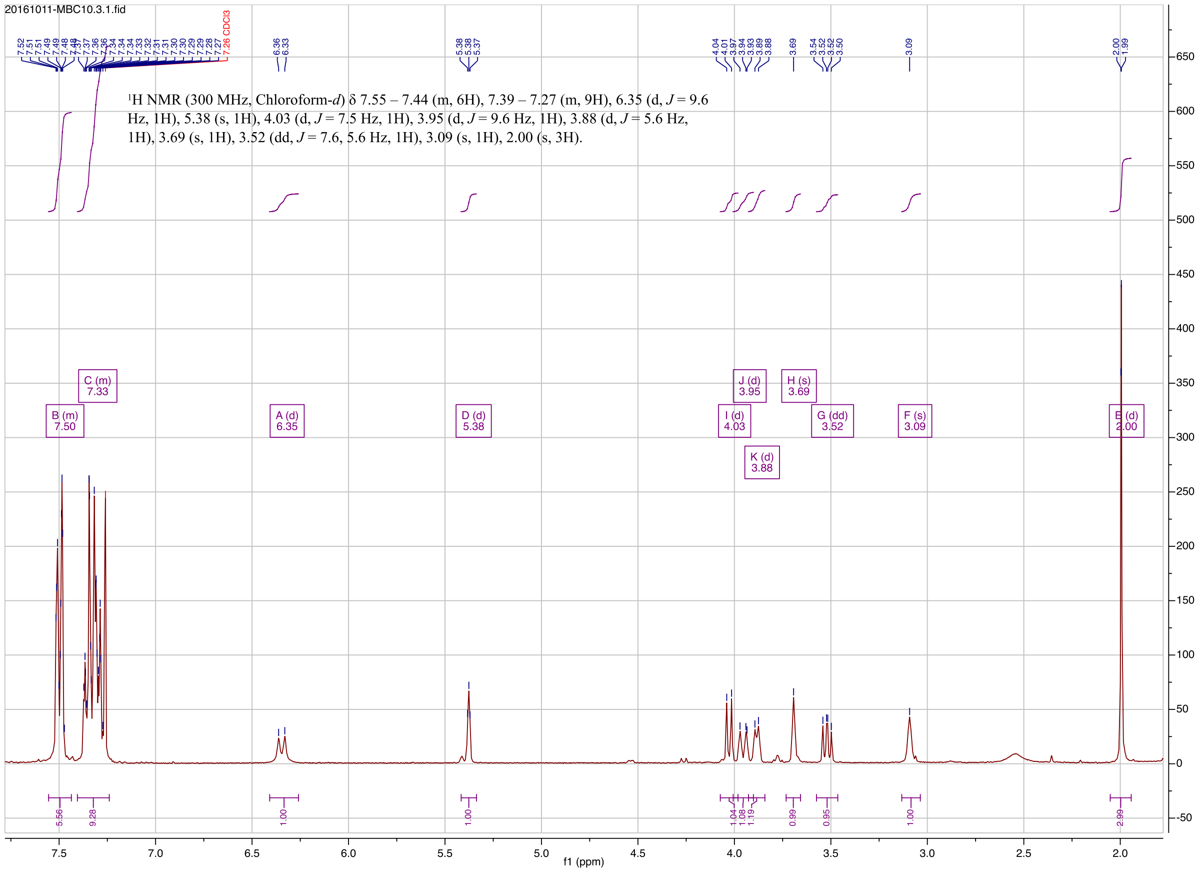

	Observed	Reported
H-1	5.38	5.40
H-2	3.95	3 973
H-3	3.09	3 080
H-4	3.69	3 705
H-5	3.88	3 910
H-6a	4.03	4 350
H-6b	3.52	3 538
NHAc	6.35	6 350
Ac	2.00	2 198
Ar	7.27-7.55	7.28-7.55

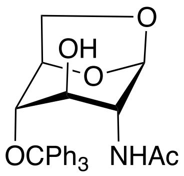

12

¹H NMR data for **12** ($CDCl_3$); Literature data from Tyrtysh, T. V.; Byramova, N. E.; Bovin, N. V. *Russian Journal of Bioorganic Chemistry,* **2000,** *26,* 414-418.

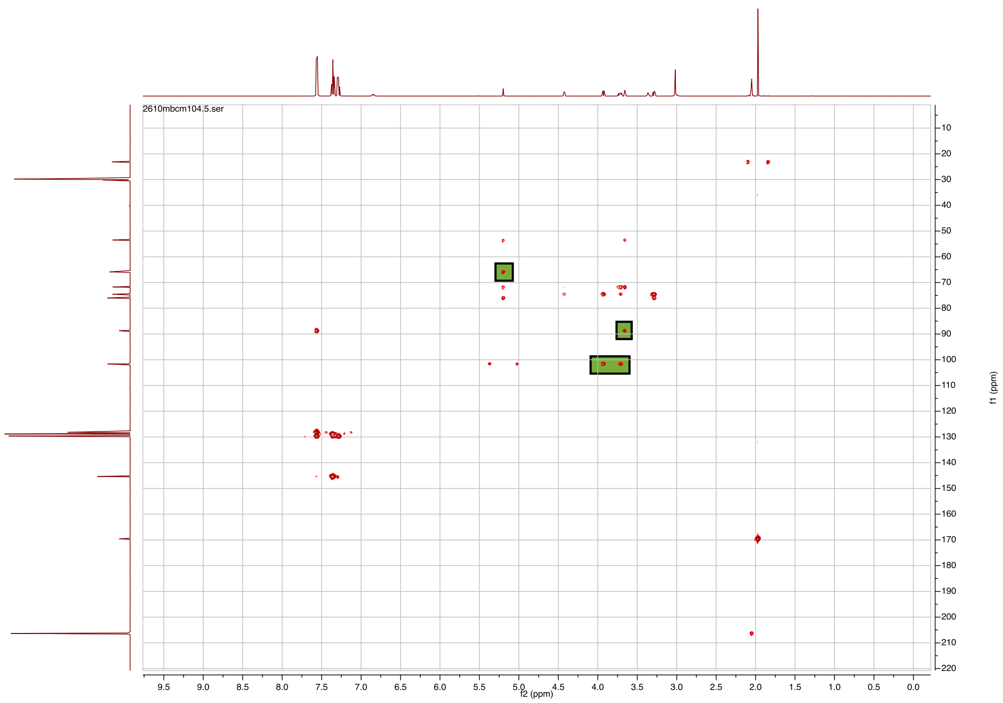

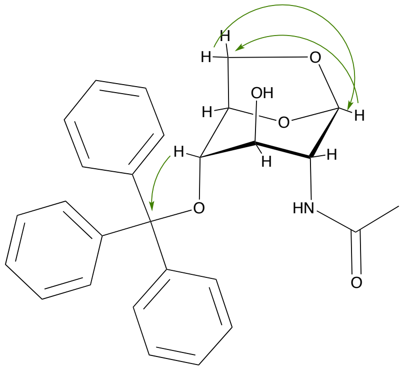

HMBC

HMBC spectrum for **12** with key correlations indicated on spectrum and structure

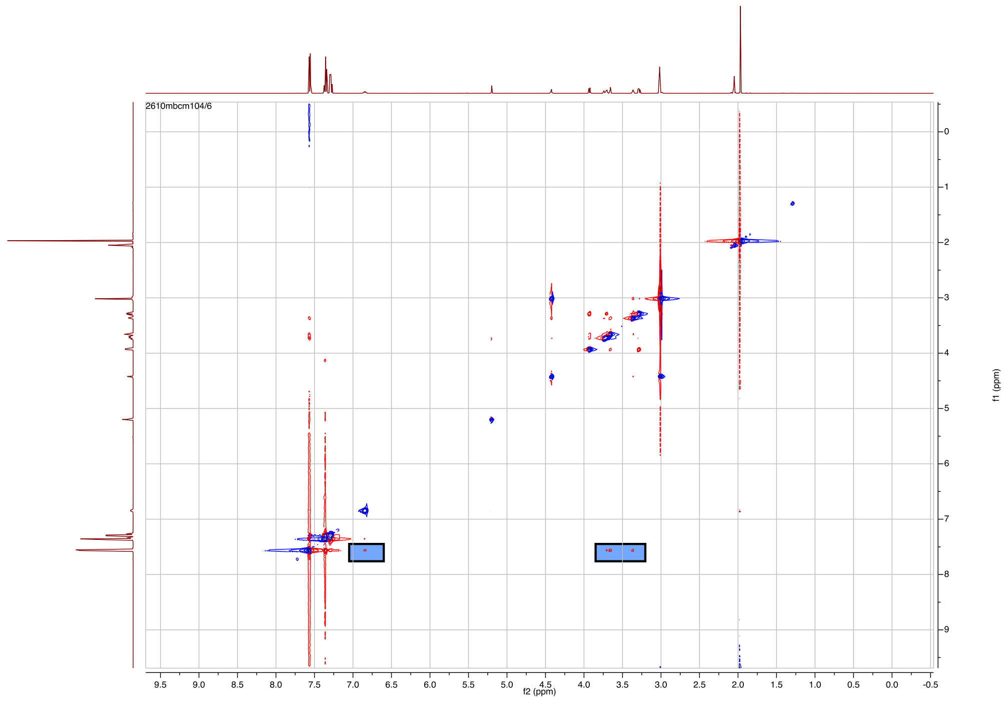

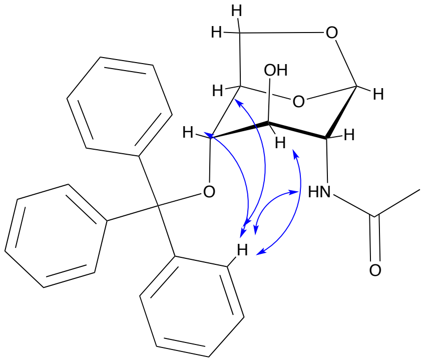

NOESY

NOESY spectrum for **12** with key correlations indicated on spectrum and structure

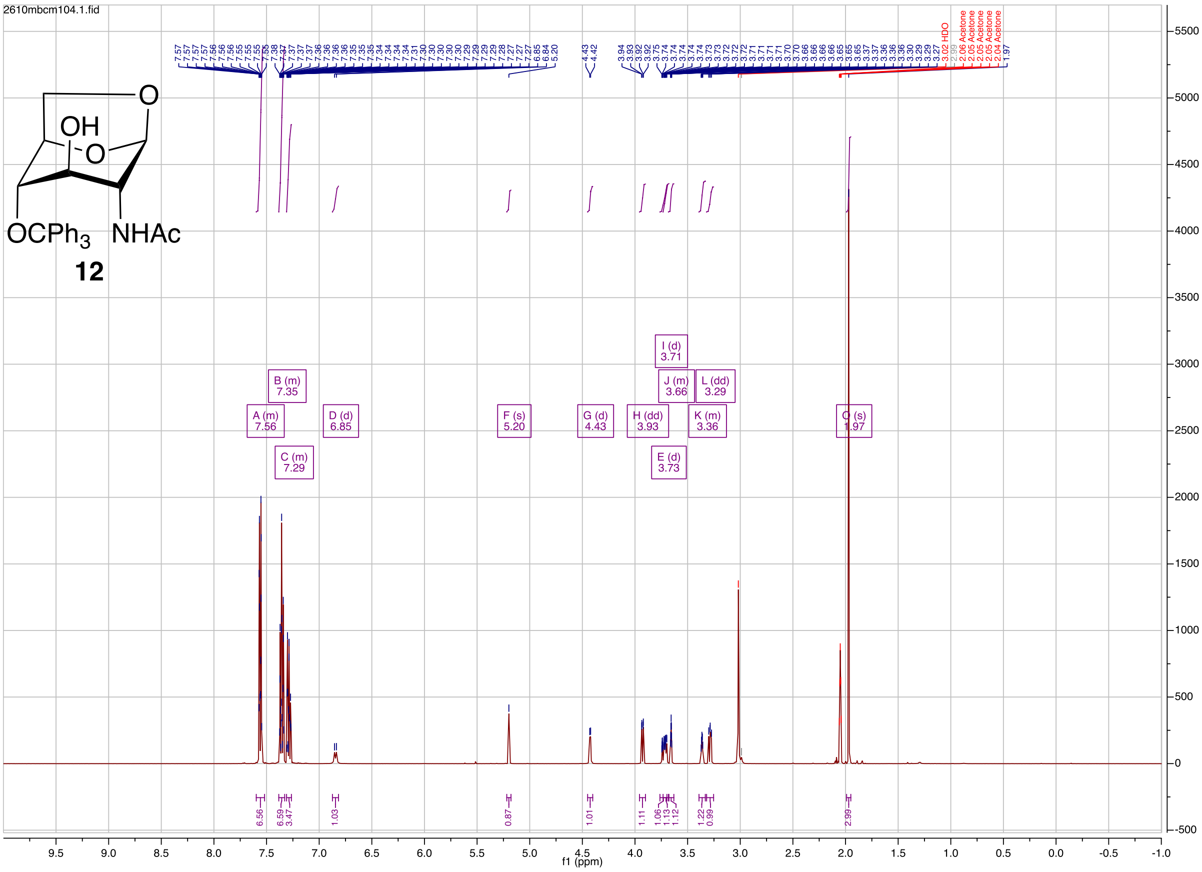

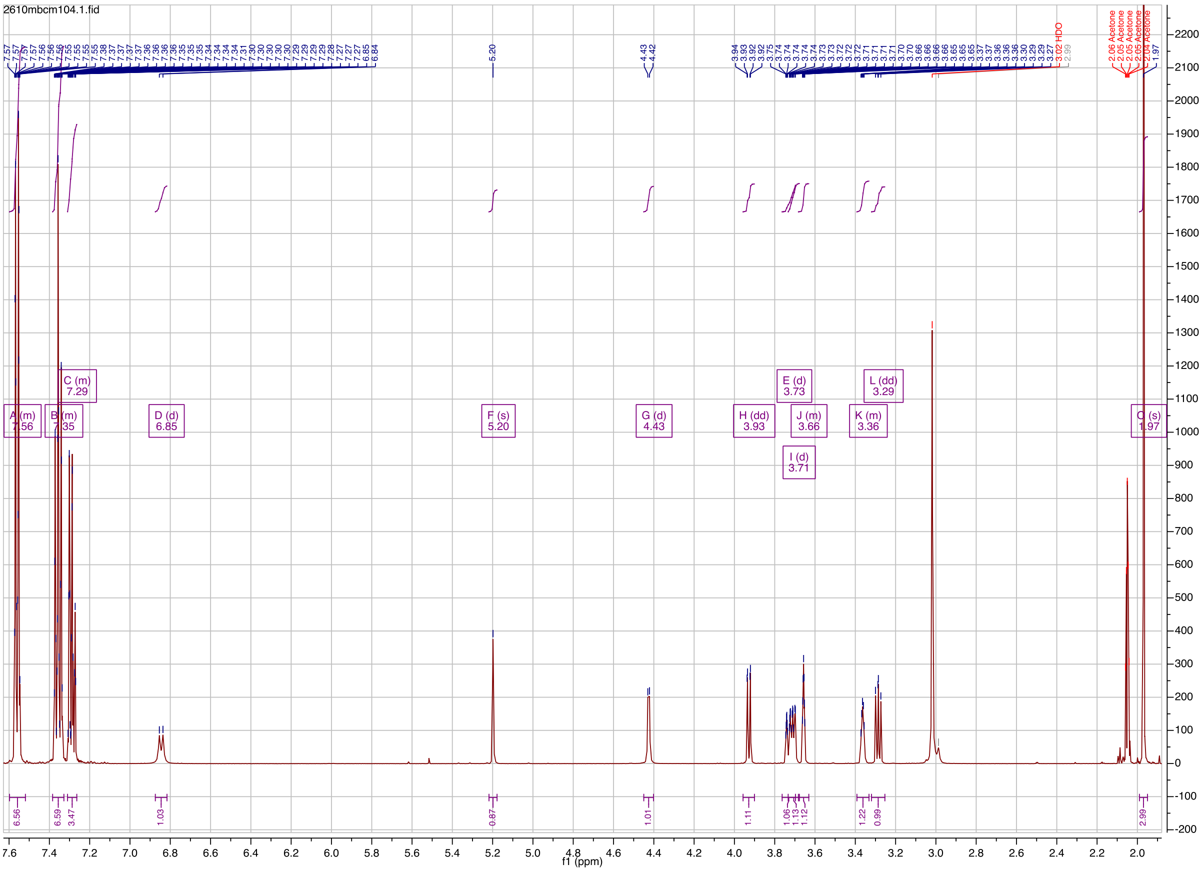

^{1}H NMR data for **12**

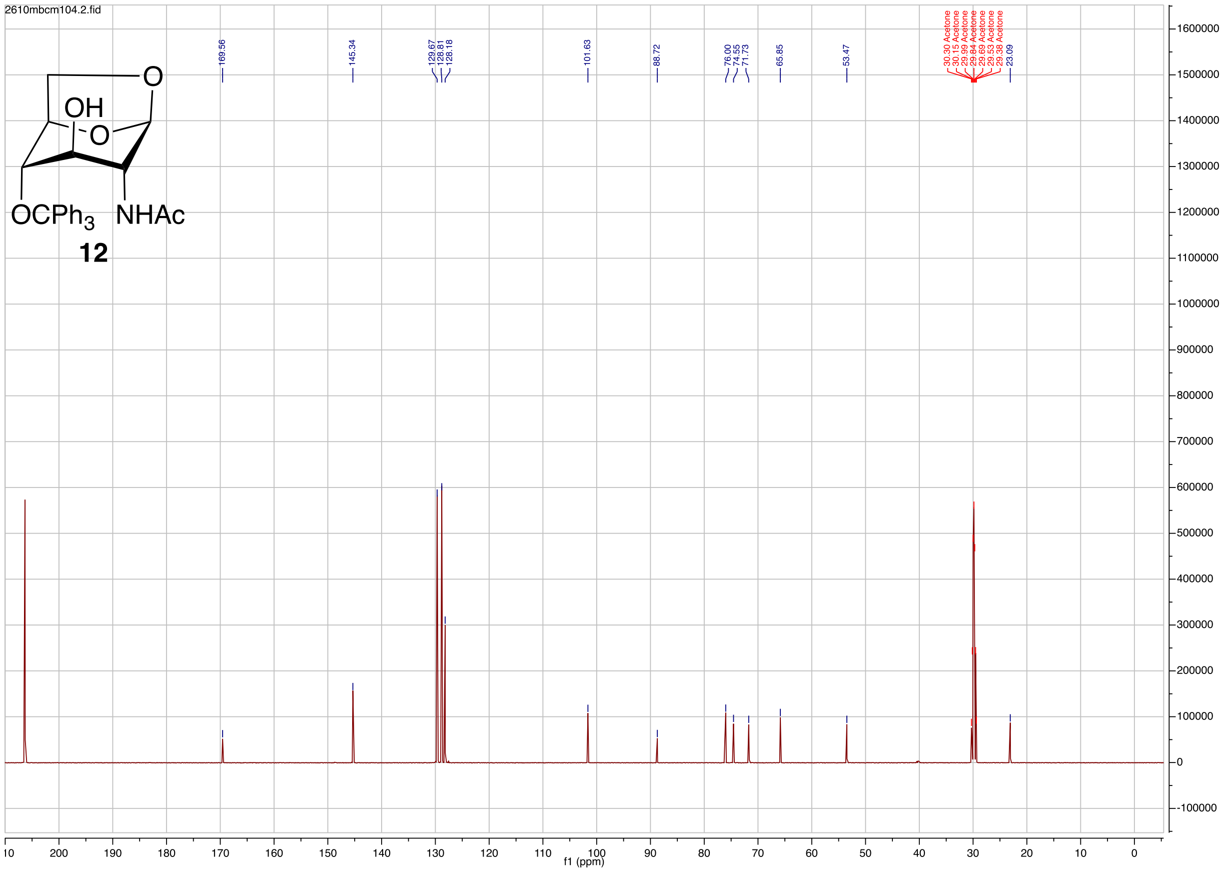

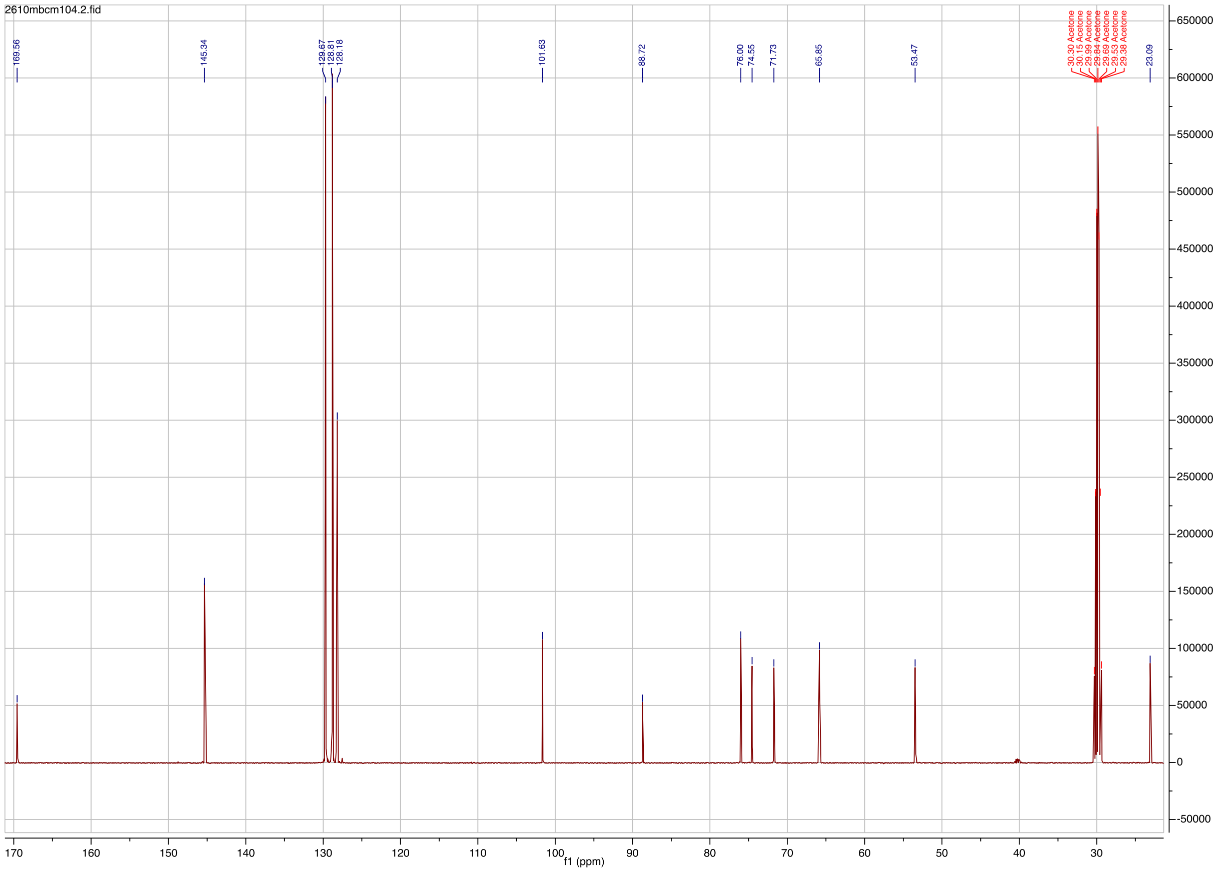

^{13}C NMR data for **12**

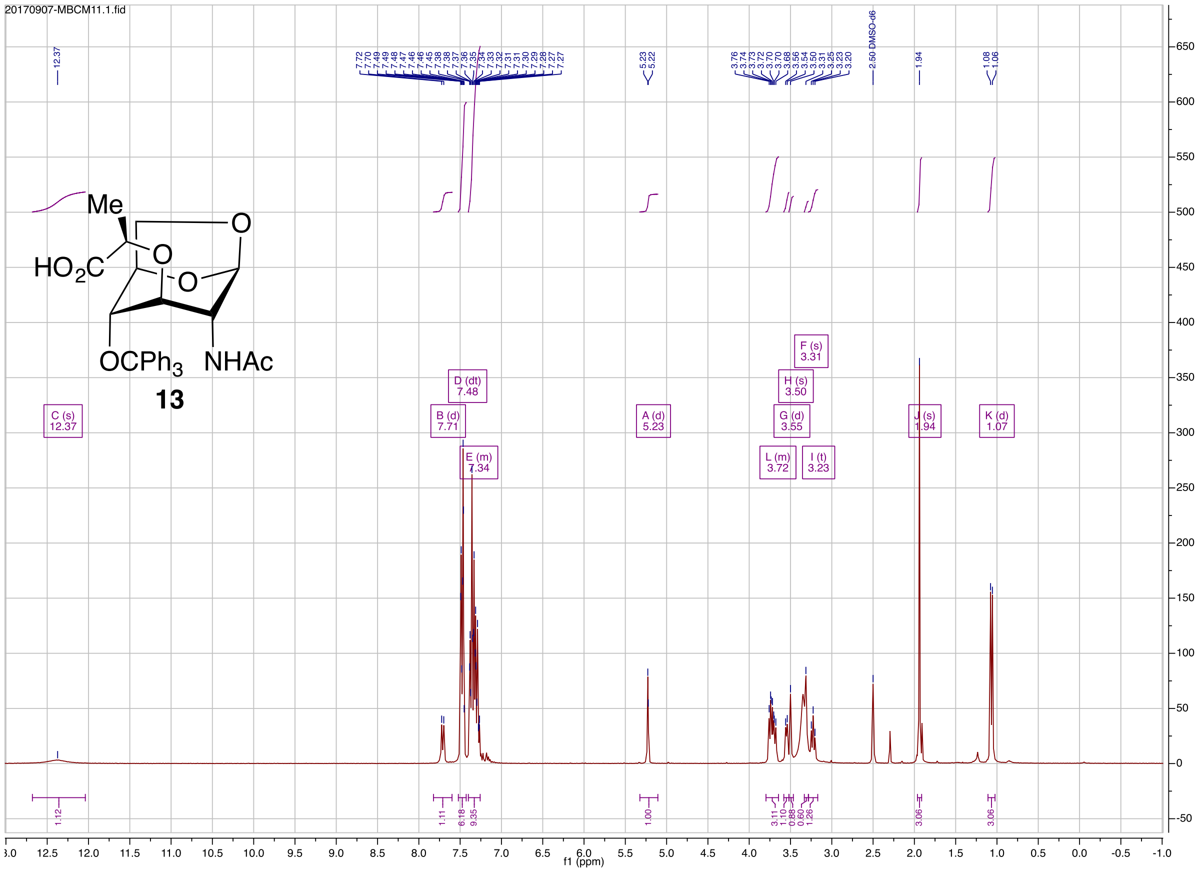

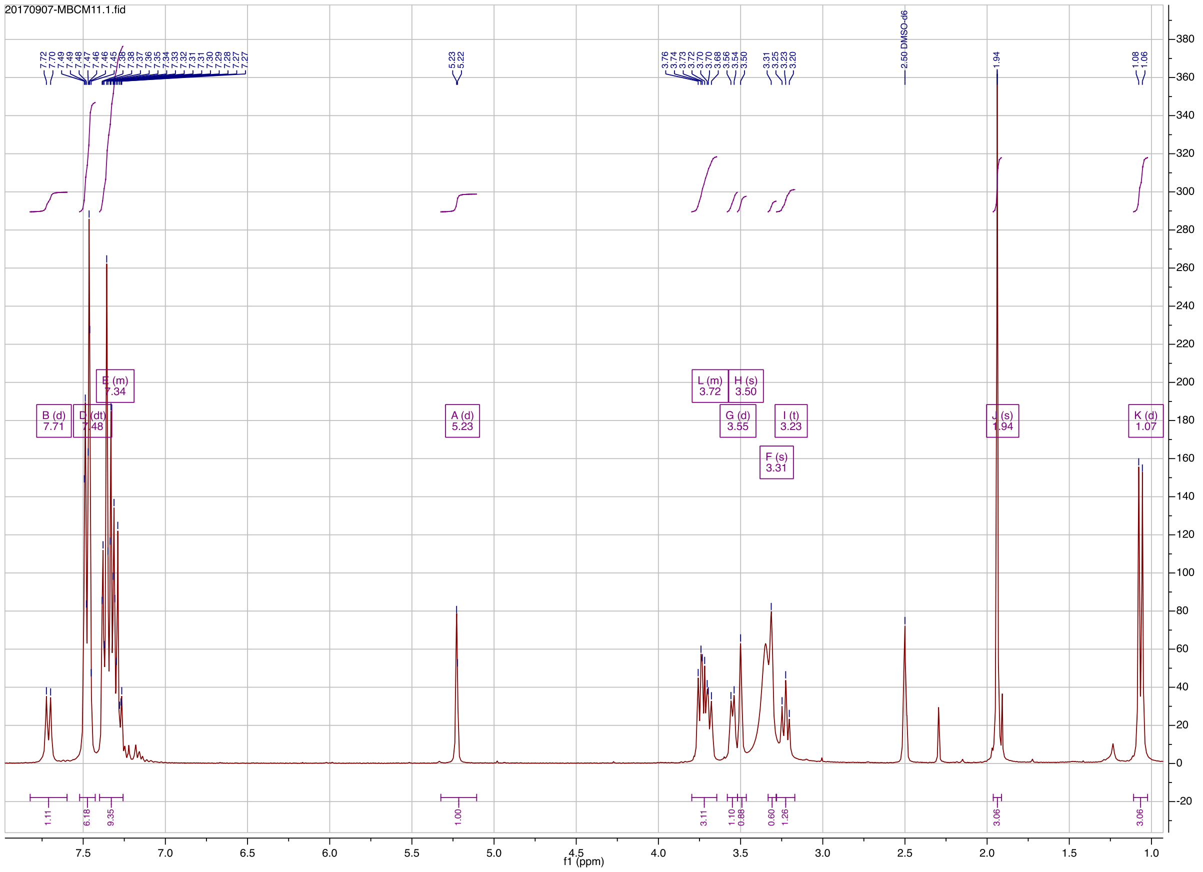

^{1}H NMR data for **13**

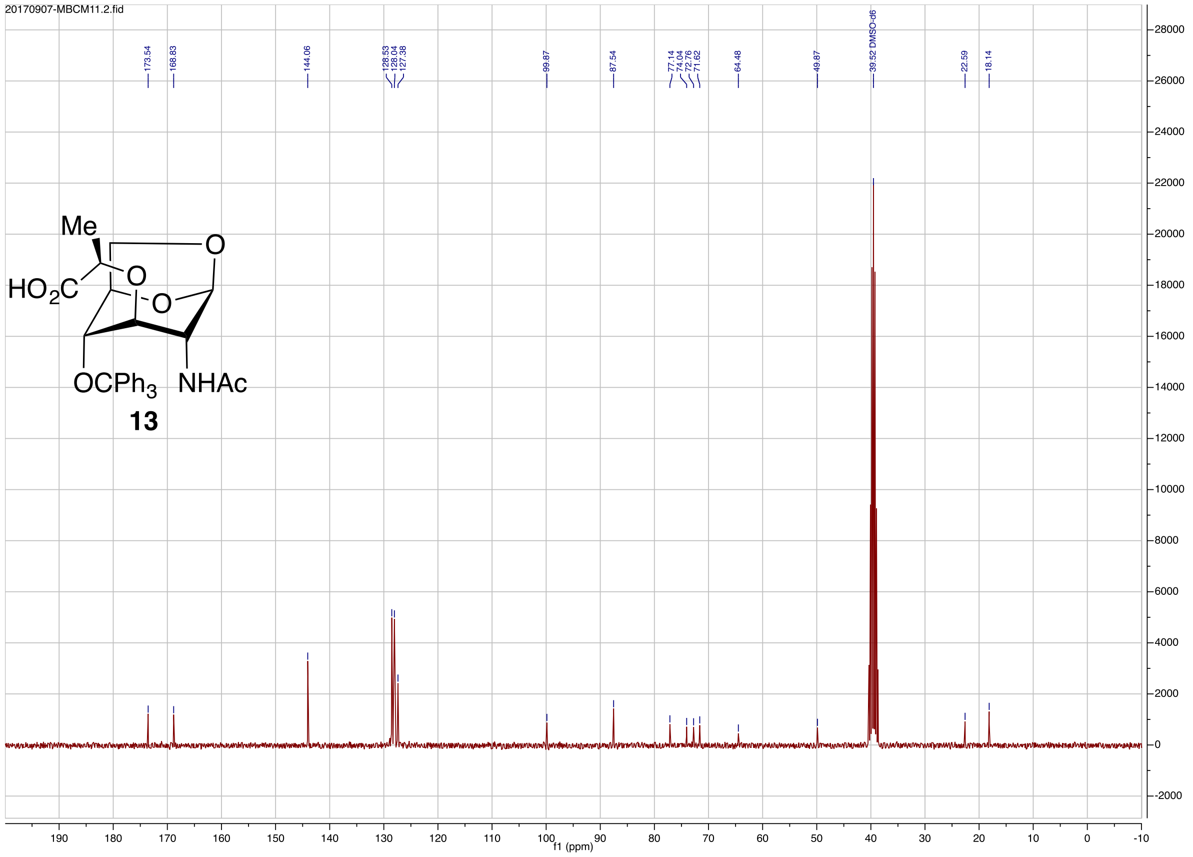

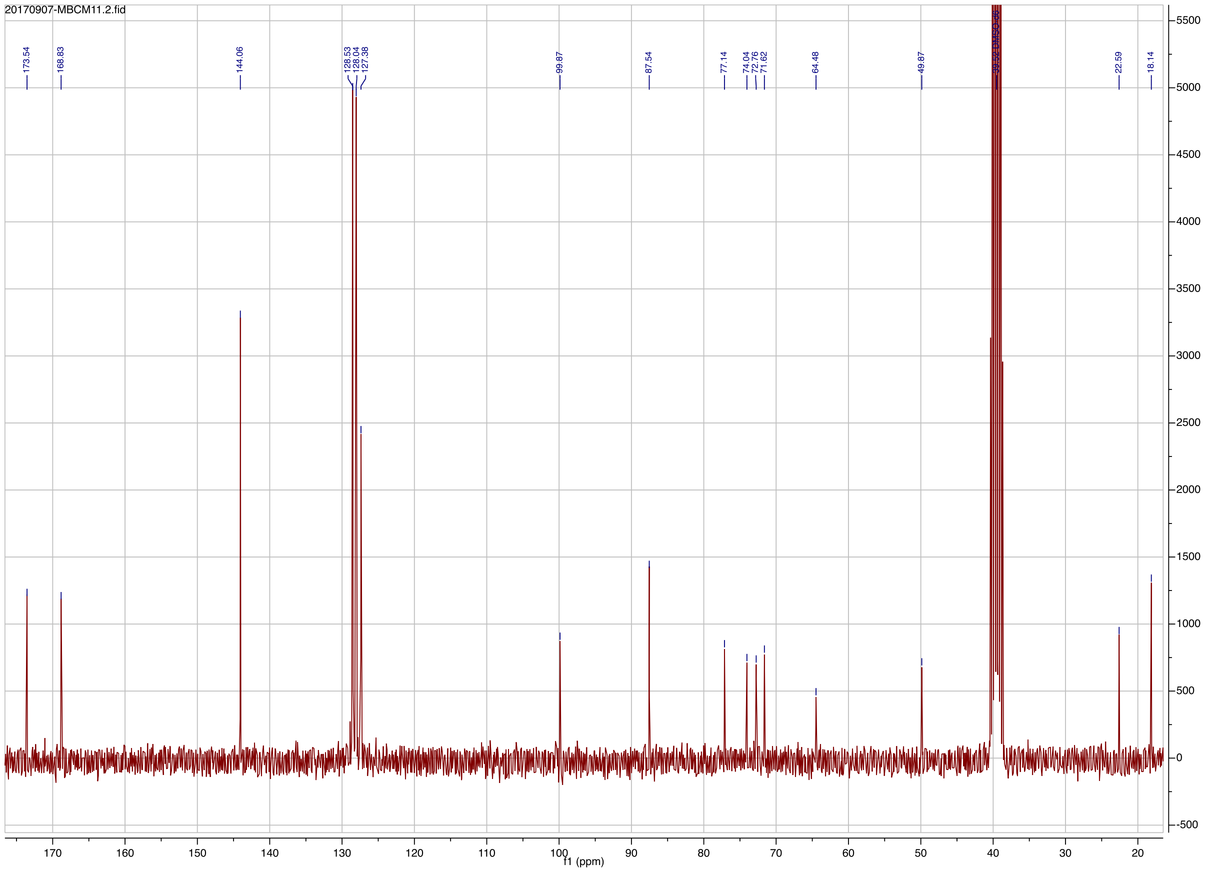

^{13}C NMR data for **13**

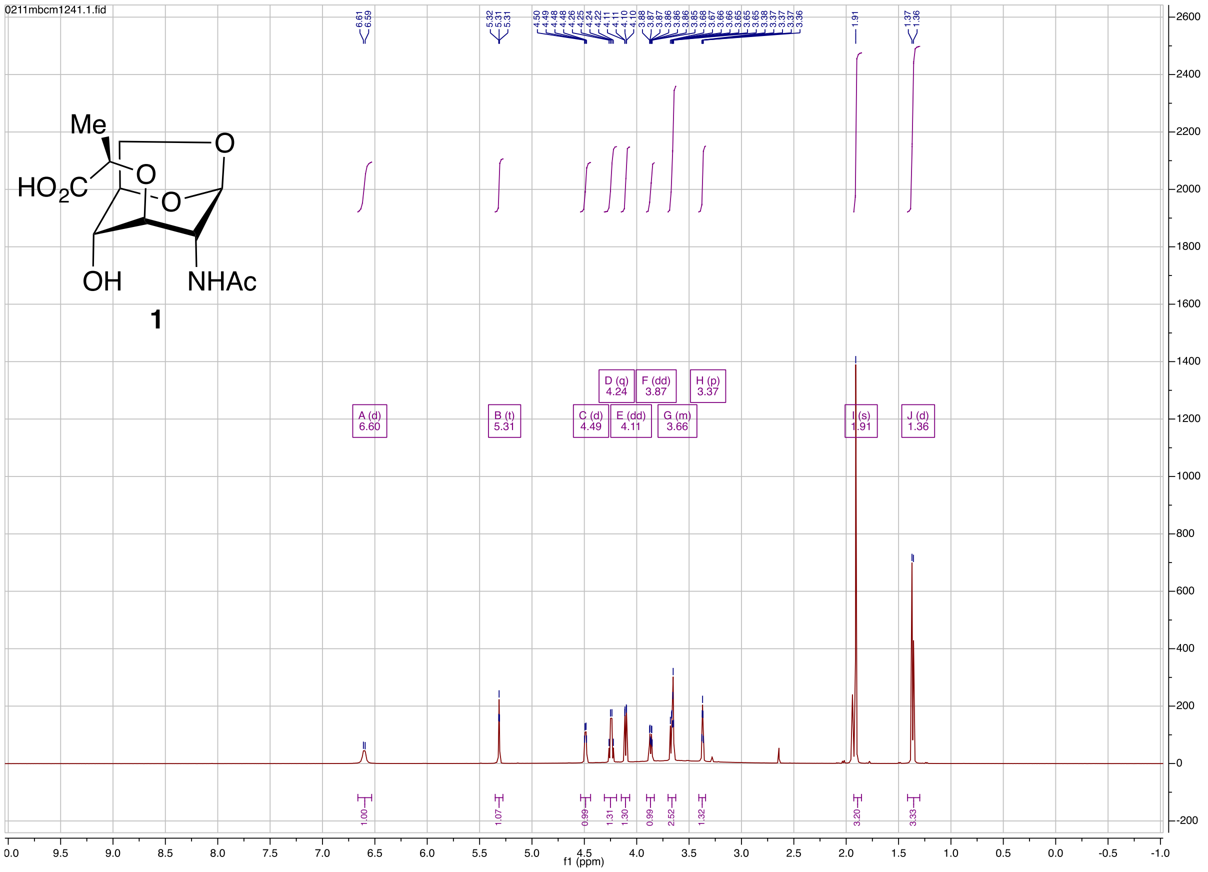

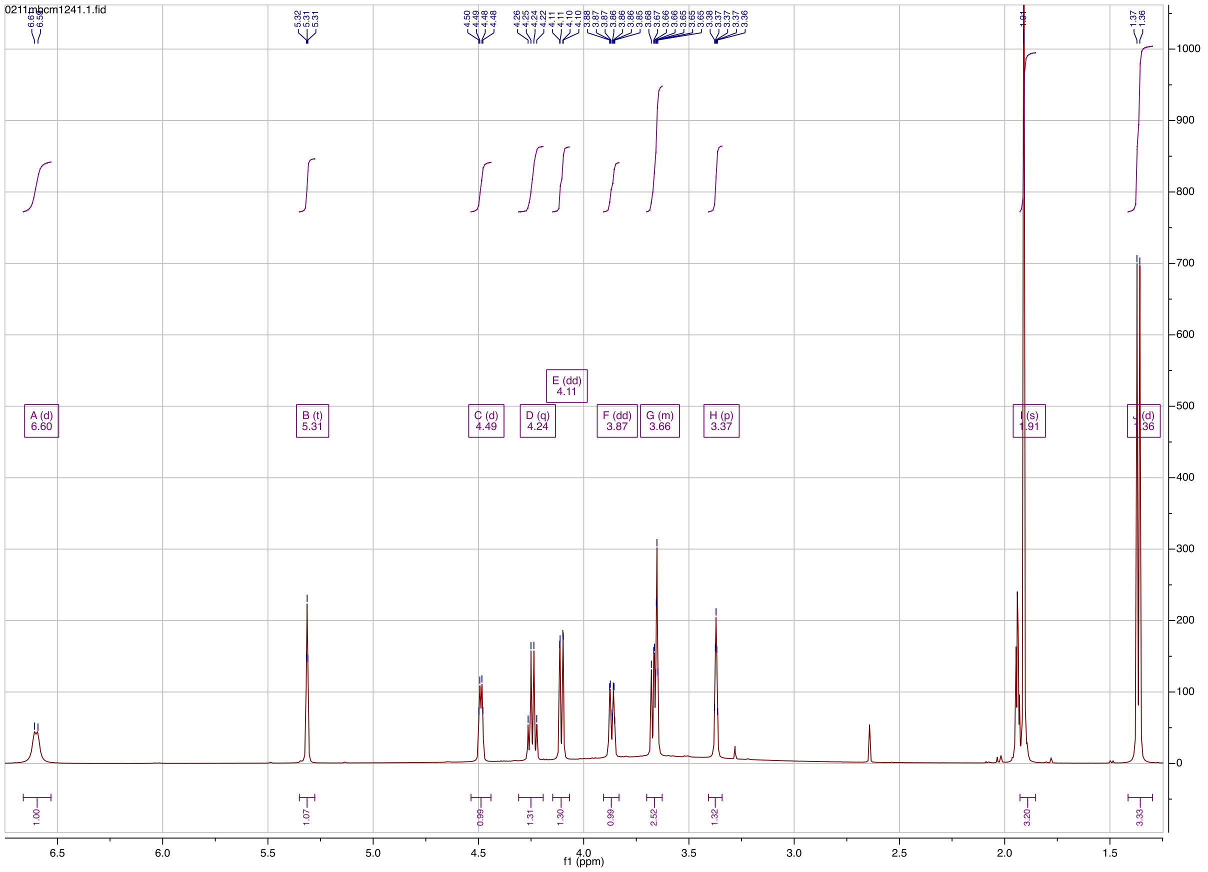

^{1}H NMR data for **1**

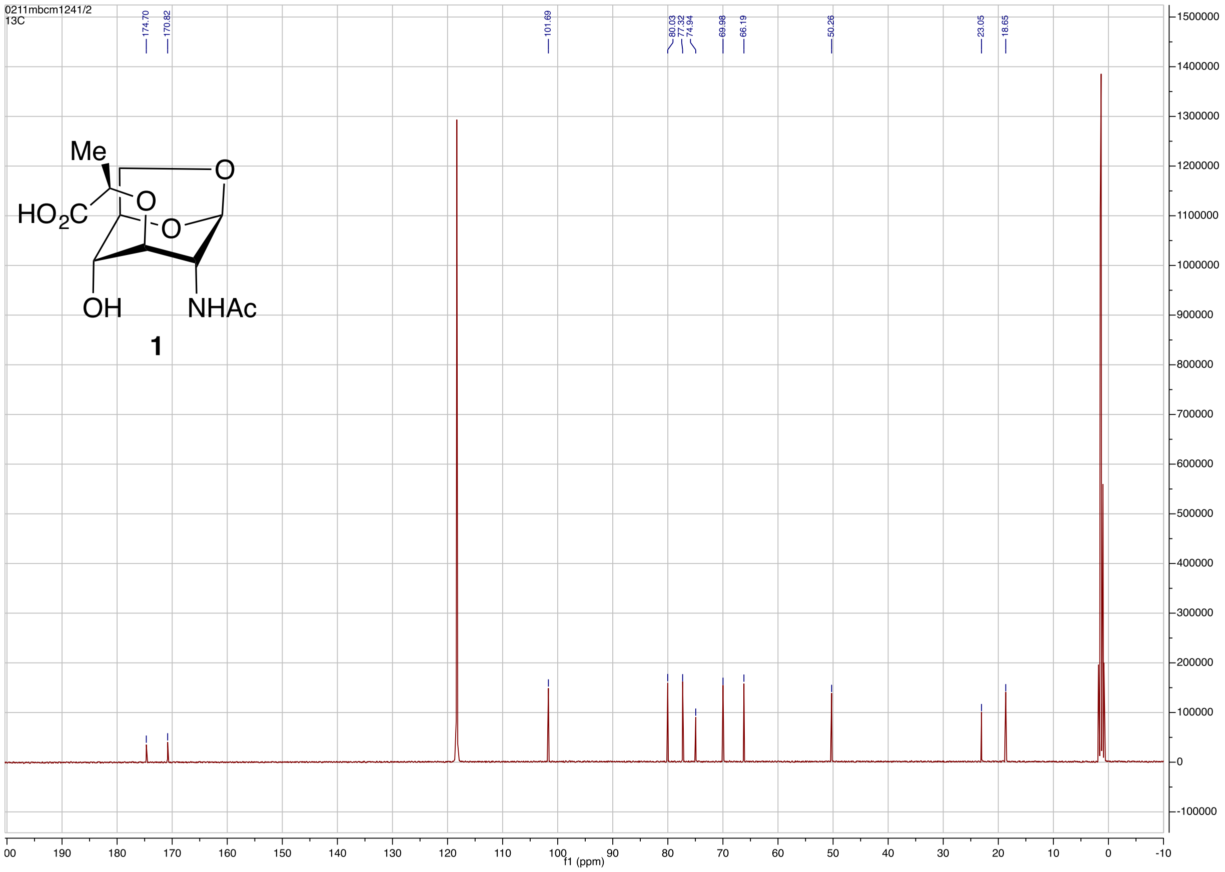

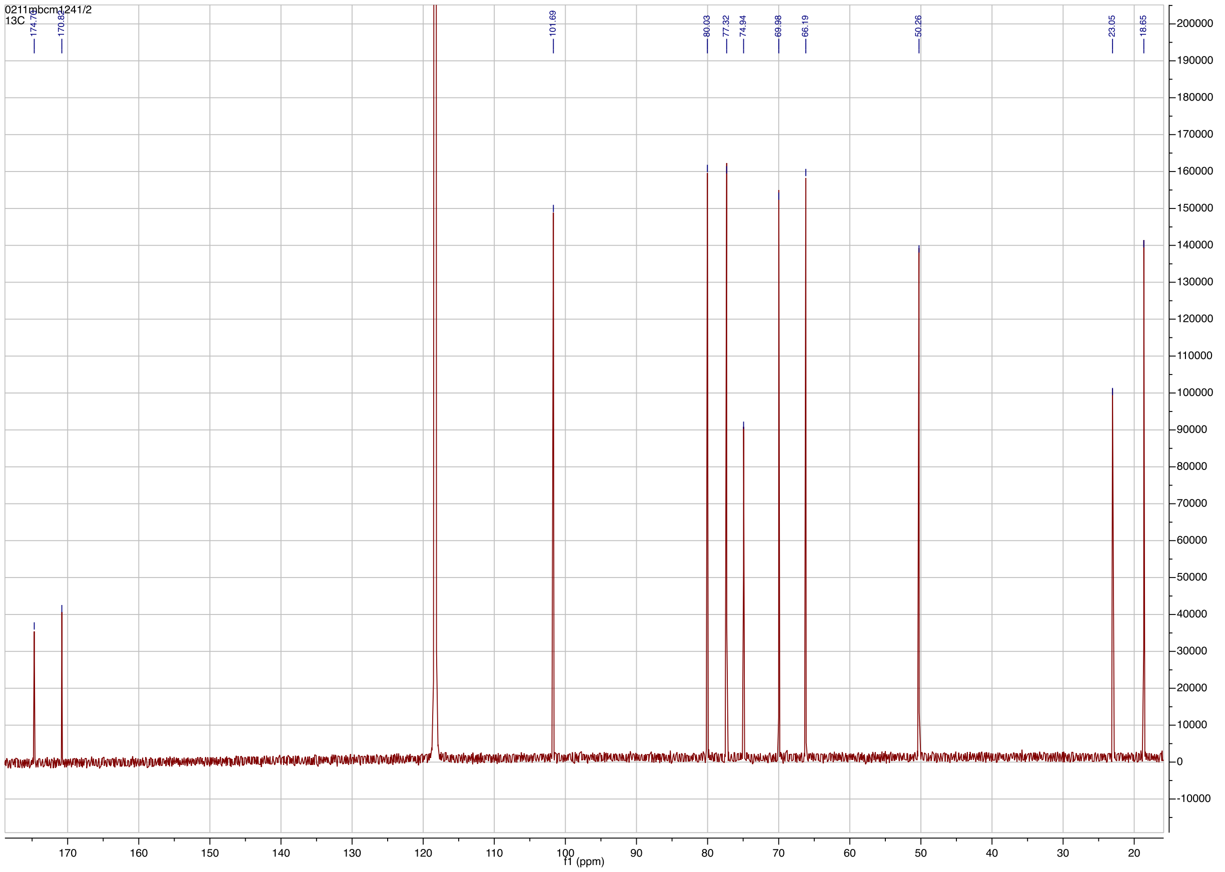

^{13}C NMR data for **1**

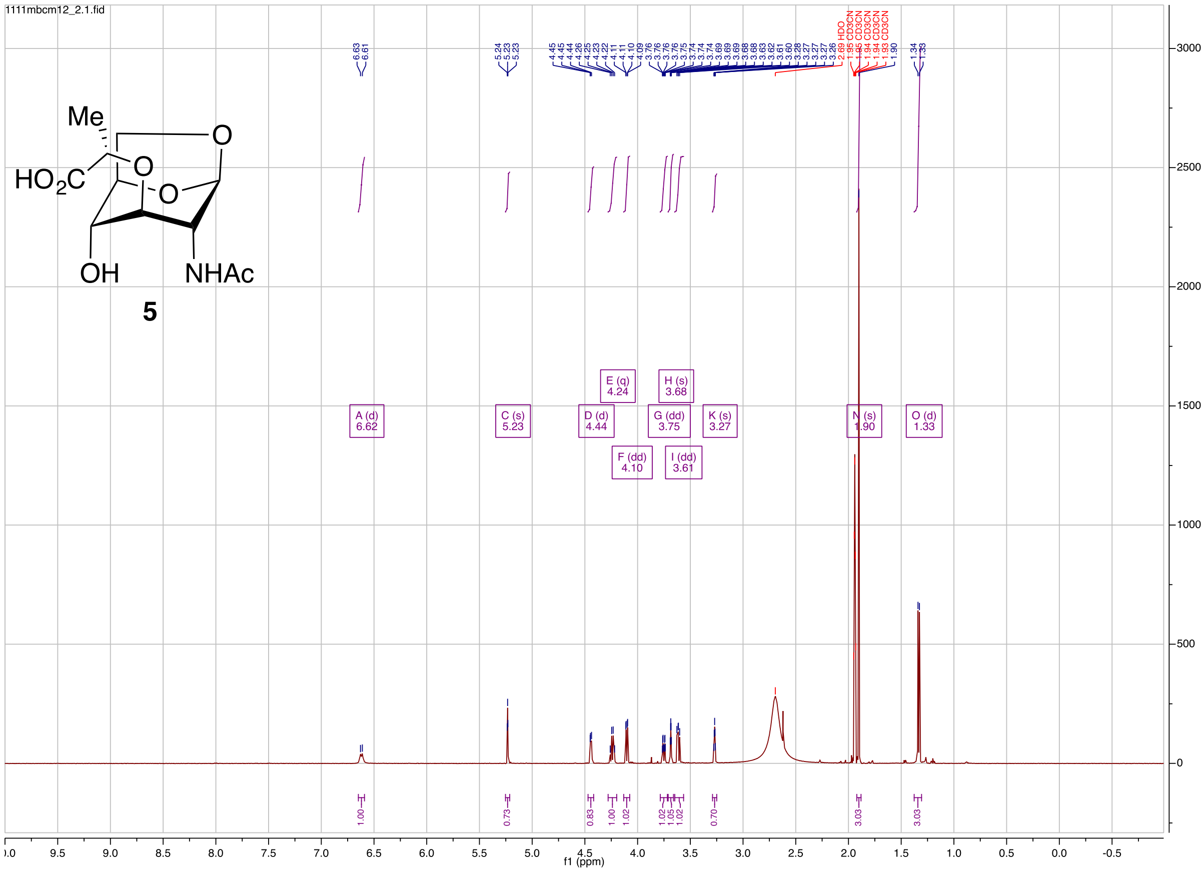

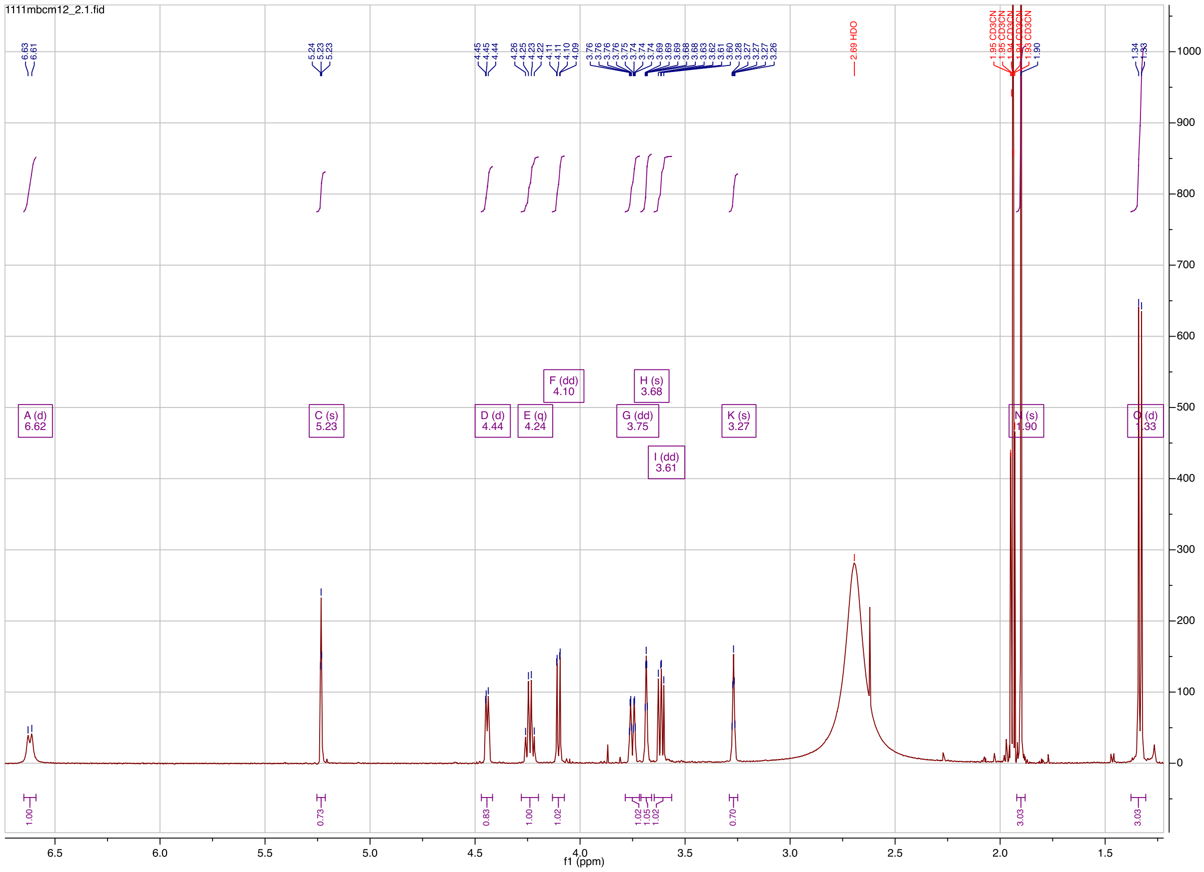

^{1}H NMR data for **5**

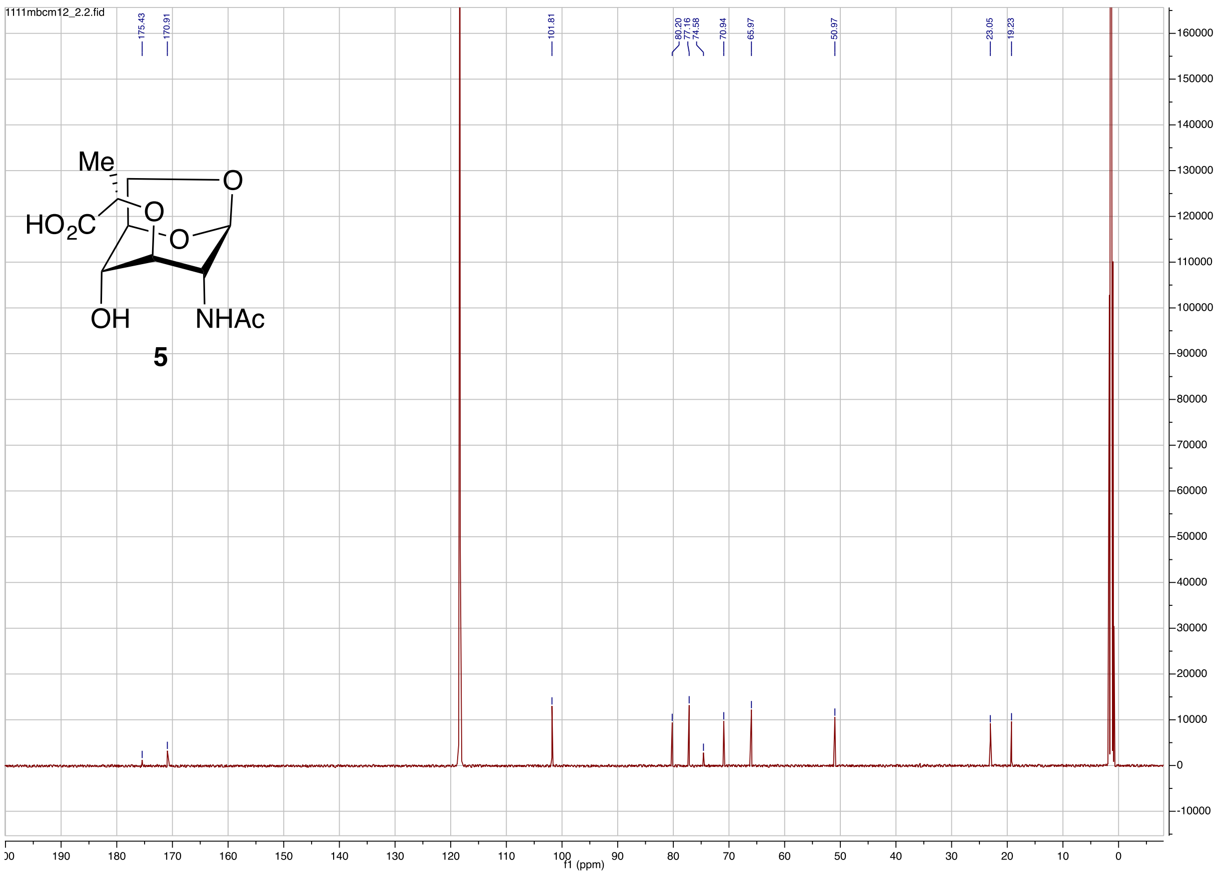

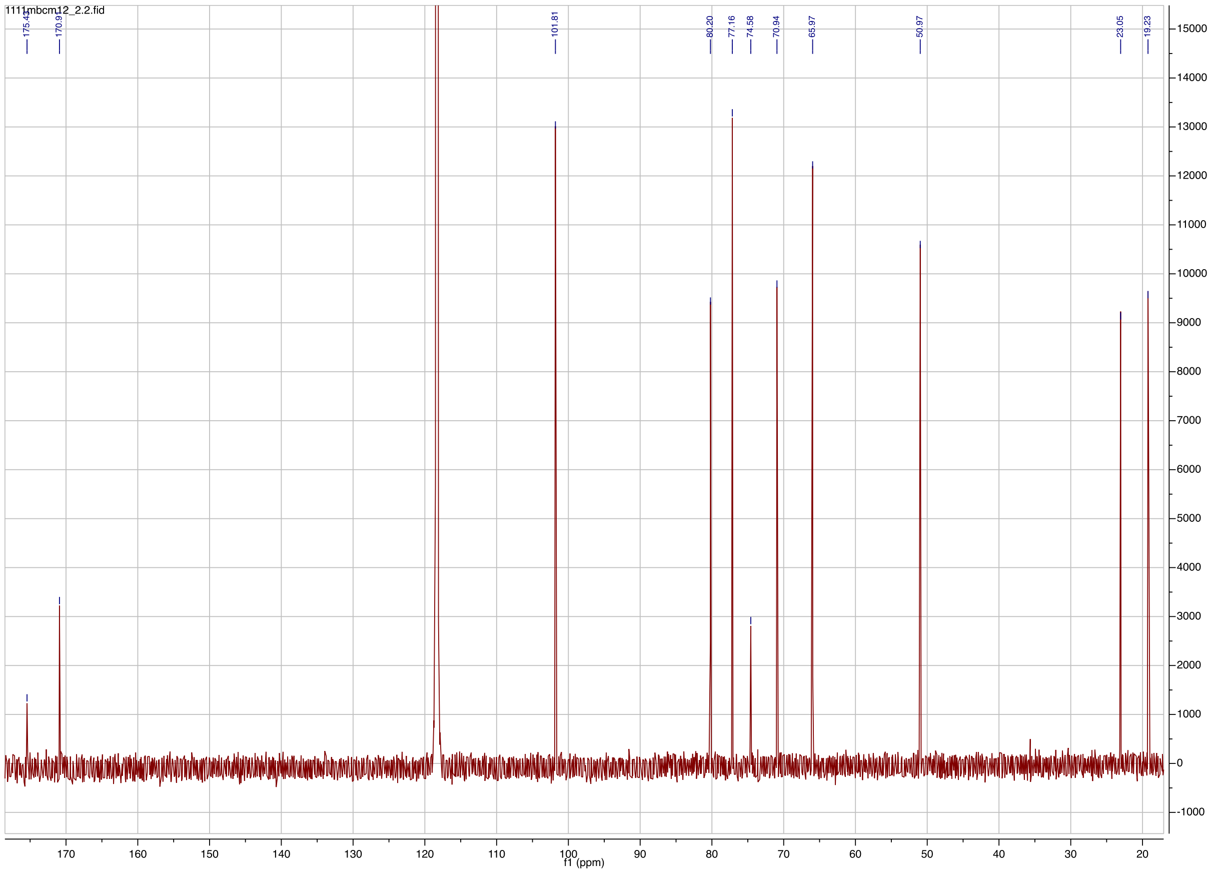

^{13}C NMR data for **5**
